# A comprehensive dataset for bibliometric analysis of SARS and coronavirus impact on social sciences

**DOI:** 10.1016/j.dib.2020.106520

**Published:** 2020-11-14

**Authors:** Kamran Shaukat, Talha Mahboob Alam, Ibrahim A. Hameed, Suhuai Luo, Jiaming Li, Gagandeep Kaur Aujla, Farhat Iqbal

**Affiliations:** 1 School of Electrical Engineering and Computing, The University of Newcastle, Callaghan, NSW 2308, Australia; 2 Department of Computer Science, University of Engineering and Technology, Lahore 54890, Pakistan; 3 Department of ICT and Natural Sciences, Norwegian University of Science and Technology, Trondheim 7491, Norway; 4 Data61, Commonwealth Scientific and Industrial Research Organization, Clayton South, VIC 3169, Australia

**Keywords:** Coronavirus, COVID-19, SARS, Pandemic, Social sciences, SARS-CoV-2

## Abstract

The year 2020 has changed the living style of people all around the world. Corona pandemic has affected the people in all fields of life economically, physically, and mentally. This dataset is a collection of published articles discussing the effect of COVID and SARS on the social sciences from 2003 to 2020. This dataset collection and analysis highlight the significance and influential aspects, research streams, and themes in this domain. The analysis provides top journals, highly cited articles, mostly used keywords, top affiliation institutes, leading countries based on the citation, potential research streams, a thematic map, and future directions in this area of research. In the future, this dataset will be helpful for every researcher and policymakers to proceed as a starting point to identify the relevant research based on the analysis of 18 years of research in this domain.

## Specifications Table

**Table:** 

Subject	Meta-analysis
Specific subject area	Healthcare
Type of data	Table Image Graph Figure
How data were acquired	Articles were screened using the electronic database search. We have also used a web crawler to identify several web references.
Data format	Raw Analyzed
Parameters for data collection	Electronic databases such as Web of Science (WOS), Emerald, and PubMed using the keywords, literature searched from 2003 to 2021.
Description of data collection	5000 articles were screened using the electronic database search, and after removing duplicates and excluding articles as per exclusion criteria, 1000 full-text articles remained for further evaluation. Finally, 1827 articles remained for final data analysis.
Data source location	Available at https://data.mendeley.com/datasets/w7kz8n3s3y/2 Dataset was collected and analyzed in Australia. Secondary data: Electronic databases; Primary data: PubMed, Embase, Cochrane
Data accessibility	Repository name: Mendeley Data Data identification number: 10.17632/w7kz8n3s3y.1 Direct URL to data: https://data.mendeley.com/datasets/w7kz8n3s3y/2
Related research article	Authors’ names: Adeel Nasir, Kamran Shaukat, Ibrahim A. Hameed, Suhuai Luo, Talha Mahboob Alam, And Farhat Iqbal Title: A Bibliometric Analysis of Corona Pandemic in Social Sciences: A Review of Influential Aspects and Conceptual Structure Journal: IEEE Access https://doi.org/10.1109/access.2020.3008733

## Value of the Data

• This data provides the list of articles published from 2003 to 2021 related to the impact of SARS and Coronavirus on Social Science.
• This data is useful for all the researchers that want to extend their research in this domain. This data provides a comprehensive analysis of top journals, authors, articles, and research streams. The streams and themes will be beneficial for policymakers, researchers, and scholars for future research.
• This data is a starting point for any researcher who wants to pursue the research in this domain. Furthermore, they can have an idea of a thematic map, research streams in this direction.

## 1 Data Description

**Fig. 1:**
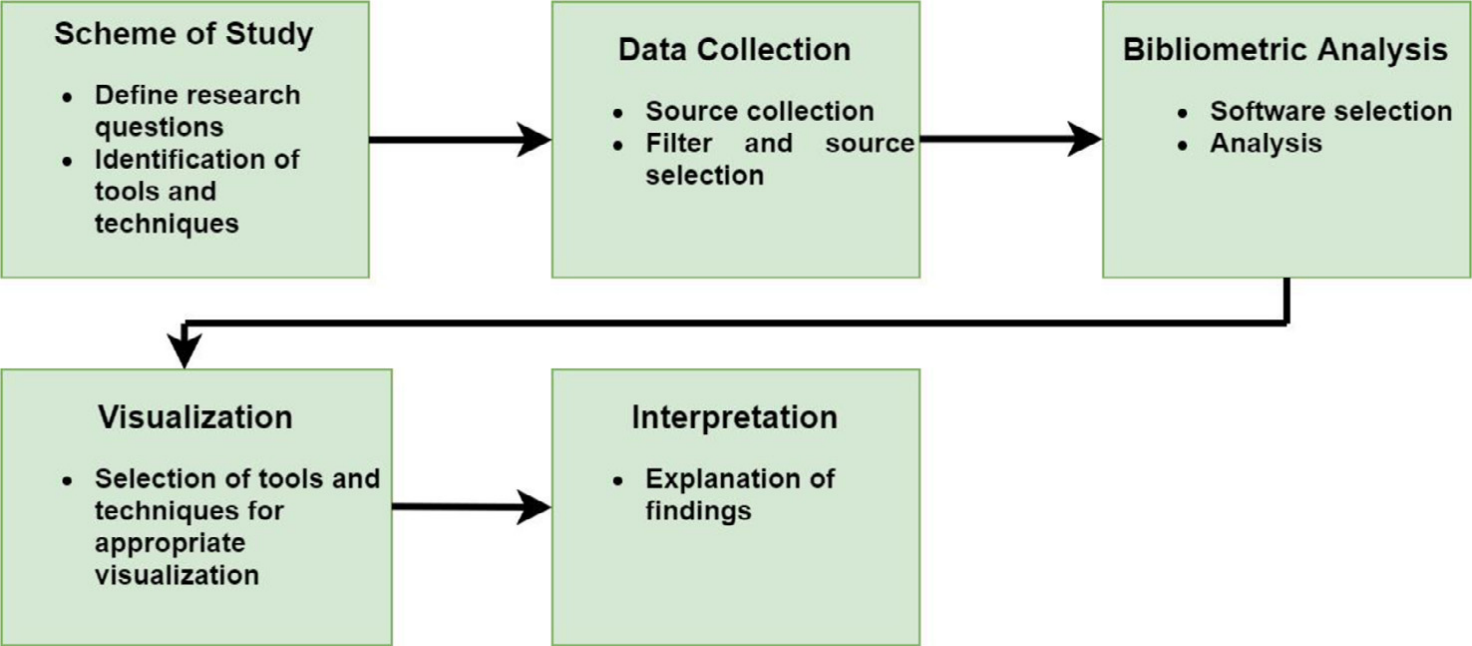
The proposed method for analysis adapted from [1].

The dataset contains the publications related to the impact of SARS and coronavirus on social sciences. This data is composed of two categories, including raw and analyzed. The raw file is available at http://dx.doi.org/10.17632/w7kz8n3s3y.2 . The analysis part is composed of tables and figures. Table 1 provides the description characteristics of coronavirus literature. The follow-up analysis is performed on 1827 articles collected from 591 multiple resources from 2003 to 2021. There were 4637 distinct authors. The collaboration index is 3.37. Table 2 provides the list of top 10 journals in this field, including the names, h-index, g-index, m-index, the total number of citations (TC), net production (NP), and starting year of the journal (PY-Start). Table 3 provides the list of most cited articles, including the count of citations and count of citations per year. Fig. 1 discusses the proposed methodology for the problem formulation.

**Table 1:** Descriptive Characteristics of Corona Literature.

Description	Results
Documents	1827
Sources (Journals)	591
Keywords Plus (ID)	2041
Author's Keywords (DE)	4938
Period	2003–2021
Average citations per documents	2.985
Authors	4637
Author Appearances	5021
Authors of single-authored documents	601
Authors of multi-authored documents	4036
Single-authored documents	630
Documents per Author	0.394
Authors per Document	2.54
Co-Authors per Documents	2.75
Collaboration Index	3.37

**Table 2:** Top Ten Journals According to Source Impact.

Top 10 Journals	h_index	g_index	m_index	TC	NP	PY_start
Sustainability (Switzerland)	8	10	2	186	142	2017
American Review of Public Administration	1	1	1	6	46	2020
Tijdschrift Voor Economische En Sociale Geografie	2	2	2	11	26	2020
Gender, Work And Organization	3	4	3	34	25	2020
Tourism Geographies	7	10	7	145	25	2020
Food Security	2	2	2	13	23	2020
Journal of Air Transport Management	2	3	2	15	23	2020
Survey Research Methods	2	2	2	8	23	2020
Transportation Research Interdisciplinary Perspectives	3	7	3	59	23	2020
European Journal of Risk Regulation	4	4	4	46	22	2020

**Table 3:** Most Globally Cited Article.

Paper	Total Citations	TC per Year
Policies and Technical Guidelines for Urban Planning of High-Density Cities–Air Ventilation Assessment (AVA) of Hong Kong [2]	266	22.1667
Responding to Global Infectious Disease Outbreaks: Lessons from Sars on the Role of Risk Perception, Communication and Management [3]	248	16.5333
Perceived Travel Risks Regarding Terrorism and Disease: The Case of Thailand [4]	177	14.75
Distinguishing Knowledge-Sharing, Knowledge-Construction, and Knowledge-Creation Discourses [5]	149	12.4167
The Impact of Crisis Events and Macroeconomic Activity on Taiwan's International Inbound Tourism Demand [6]	120	10
Representations of SARS in the British Newspapers [7]	107	6.2941
Within the Boundaries of Politics: News Framing of Sars in China and the United States [8]	100	6.25
The Airborne Transmission of Infection between Flats in High-Rise Residential Buildings: Particle Simulation [9]	99	7.6154
Assessing Impacts of Sars and Avian Flu on International Tourism Demand to Asia [10]	93	7.1538
Pandemics, tourism and global change: A rapid assessment of COVID-19 [11]	88	88

**Fig. 2:**
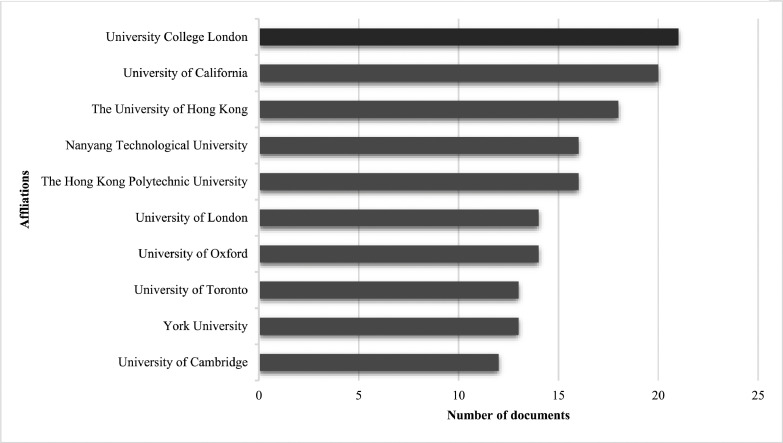
Most Relevant Affiliations.

**Fig. 3:**
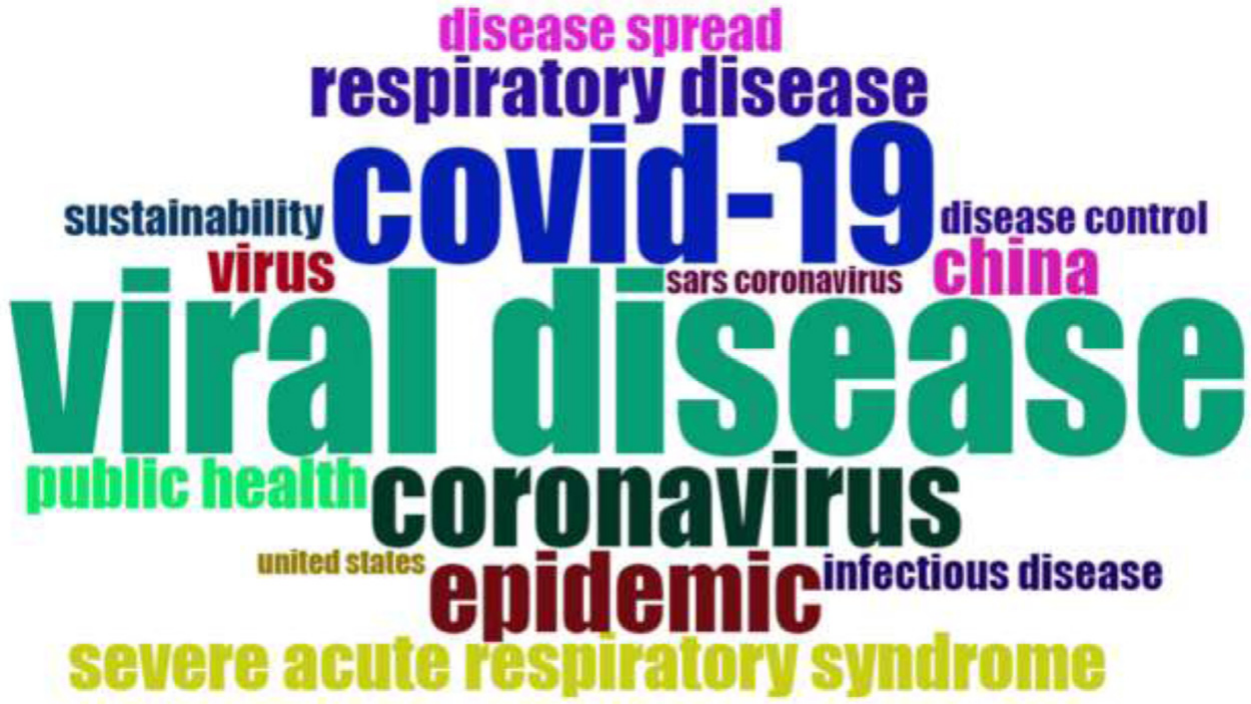
Word Cloud.

**Fig. 4:**
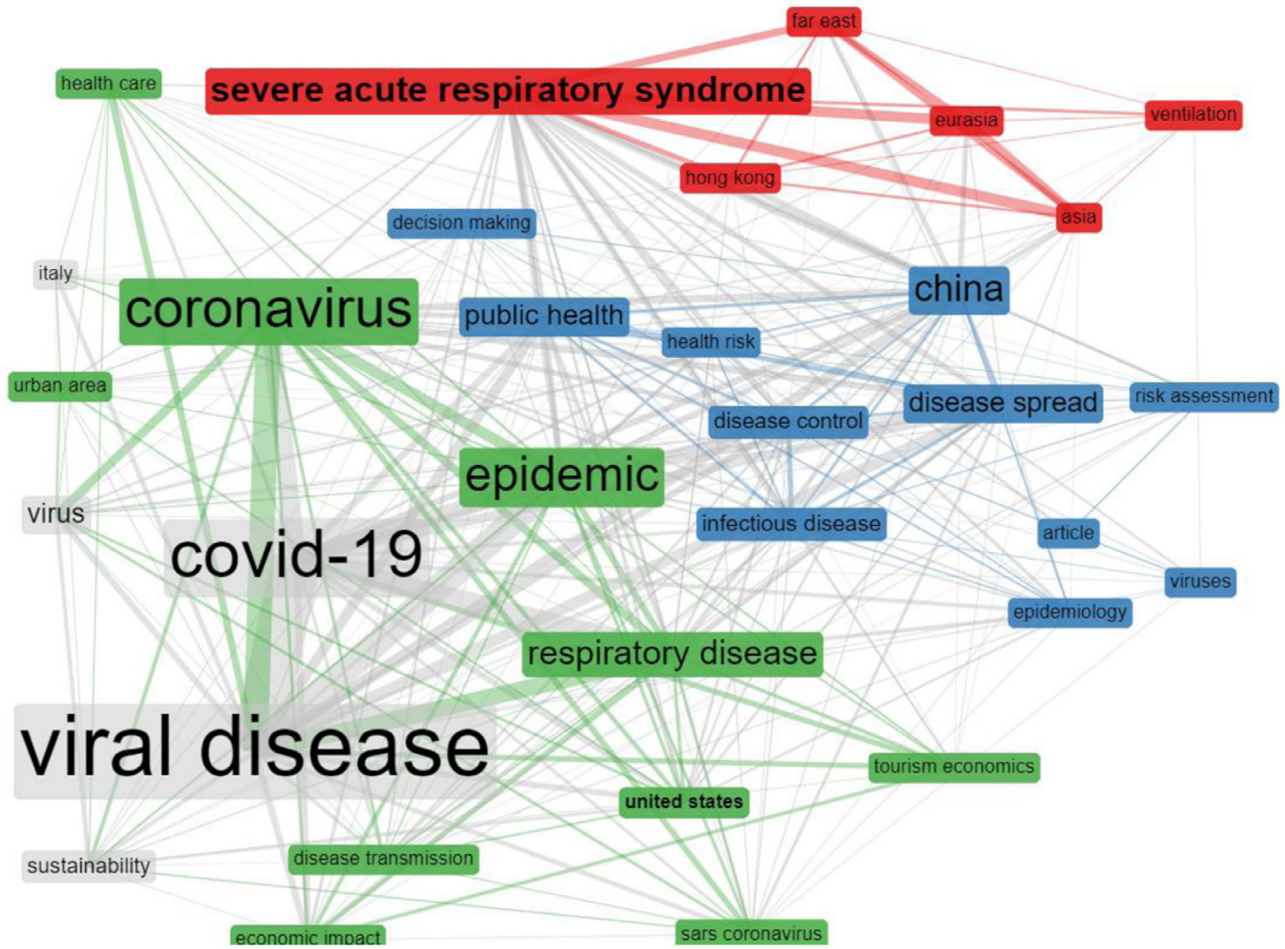
Co-occurrence network.

Fig. 2 depicts the top ten affiliations worldwide working in this area, and the *x*-axis shows the number of publications. Fig. 3 presented the word cloud of keywords plus. The larger in size the word shows, the more it occurred in literature. Fig. 4 shows the c-occurrence network of keywords used by the authors. Fig. 4 shows that the literature is divided into six different clusters. The clusters are linked with each other concerning the centrality, in terms of themes, and research streams.

## 2 Experimental Design, Materials and Methods

The coronavirus has caused economic and social damages to the whole world [12]. It is essential to see the impact of these infectious diseases on the economy and society imperative. This study aims to gather the previous literature related to infectious diseases into account and provide a biblometric analysis of the impact of coronavirus on social sciences. Multiple databases and repository were queried to get the relevant data. The search string was “corona virus" OR "corona-virus" OR "COVID19" OR "COVID-19" OR "SARS-CoV 2" OR "SARS" OR "SARS coronavirus". We have set the limit on publications year from 2003 to 2021. The data was collected on October 01, 2020. We have identified 1827 relevant articles to proceed further with analysis. This holistic analysis will enhance the literature review by provided and transparent and reproducible analysis. We have used biblioshiny tool to perform the analysis. The biblioshiny is the web interface of bibliometrix (R package). The detailed bibliometric analysis and comprehensive bibliography can be found in [1].

## Ethics Statement

The authors declare that they have no known competing financial interests or personal relationships which have, or could be perceived to have, influenced the work reported in this article.

## Supplementary Materials

Supplementary material associated with this article can be found in the online version at doi:10.17632/w7kz8n3s3y.2.

## Declaration of Competing Interest

None.
